# Supplementary material for: Brimonidine Modulates the ROCK1 Signaling Effects on Adipogenic Differentiation in 2D and 3D 3T3-L1 Cells
Source: Bioengineering (Basel). 2022 Jul 19;9(7):327. doi: 10.3390/bioengineering9070327 (PMC9311963; doi:10.3390/bioengineering9070327)
Supplement: Supplementary file 1 [file bioengineering-09-00327-s001.zip › bioengineering-1781050-supplementary.pdf]

bioengineering

MDPI

Supplementary Materials

Supplemental Table S1. Primers for qPCR.

Gene	Forward primer (5' to 3')	Reverse primer (5' to 3')	TaqMan probe (5' to 3')
**Mouse**			
36b4	TTATAACCCTGAAGTG CTCGAC	CGCTTGTACCCATT-GATGATG	/FAM/AG-GCCCTGC/ZEN/ACTCTCGCTT/IABkFQ/
Col1a1	CGCAAAGAGTC-TACATGTCTAGG	CATTGTG-TATGCAGCTGACTTC	/FAM/CCGGAGGTC/ZEN/CACAAA-GCTGAACA/IABkFQ/
Col4a1	TCTGGCTGTG-GAAAATGTGA	AATCCAATGACAC-CTTGCAAC	/FAM/TCTTTCTCC/ZEN/CTTTT-GTCCCTTCACGC/IABkFQ/
Col6a1	CCAGATGAGTGTGA-GATCCTG	AAGTTCTG-TAGGCCAATGCTC	/FAM/ACCCATTGA/ZEN/CATCCTCTTCGT GCTG/IABkFQ/
Fn1	GAGCTATCCATTTCAC-CTTCAGA	TTGTTCGTAGACAC-TGGAGA	/FAM/CAGGAGATT/ZEN/TGTTAGGAC-CACGGCA/IABkFQ/
Pparg	CTGCTCCACAC-TATGAAGACAT	TGCAGGTTCTACTTT-GATCGC	/FAM/AGCTGACCC/ZEN/AATGGTT-GCTGATTACA/IABkFQ/
Fabp4	AAATCACCGCAGAC-GACAG	CCTTTCATAACACATT CCACCAC	/FAM/TGAAGAGCA/ZEN/TCATAACCCTA-GATGGCG/IABkFQ/

Supplemental Table S2. the use of macro for Image J to define 3D spheroid size.

Image J macro

```
title = getTitle();
run("Make Composite");
run("Split Channels");
close("C3-" + title);
selectWindow("C1-" + title);
run("Duplicate...");
setAutoThreshold("Huang dark");
//run("Threshold...");
setOption("BlackBackground", true);
run("Convert to Mask");
run("Set Measurements...",
  "area mean standard integrated limit display redirect=" +   "C2-" + title + " decimal=2");
run("Analyze Particles...", "size=50000-Infinity pixel show=Outlines display exclude include");
```

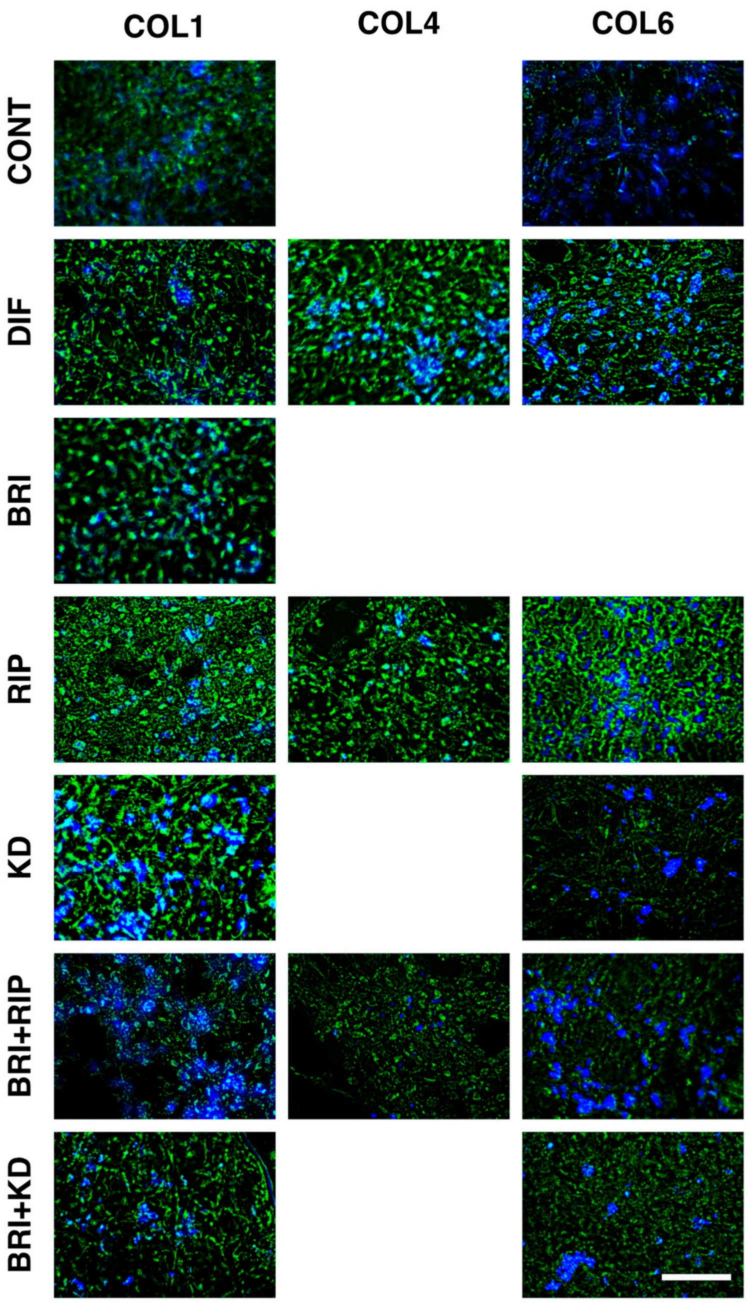

Supplemental Figure S1. Effects of ROCK-i and/or BRI on the immunolabeling of ECM proteins of 2D cultured 3T3-L1 cells.

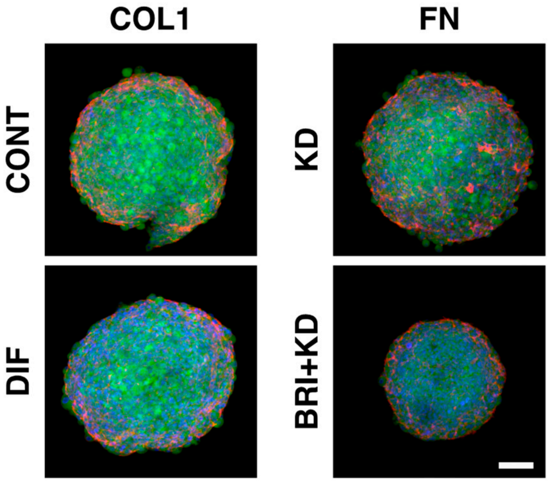

Supplemental Figure S2. Effects of ROCK-i and/or BRI on the immunolabeling of ECM proteins of 3D cultured 3T3-L1 cells.
